# Fifty Years On: GWAS Confirms the Role of a Rare Variant in Lung Disease

**DOI:** 10.1371/journal.pgen.1003768

**Published:** 2013-08-22

**Authors:** Alice M. Turner

**Affiliations:** 1 University of Birmingham, QEHB research labs, Birmingham, United Kingdom; 2 Heart of England NHS Foundation Trust, Birmingham, United Kingdom; Dartmouth College, United States of America

This year, 2013, is the fiftieth anniversary of the discovery of alpha one antitrypsin deficiency (AATD), a disease caused by mutation in *SERPINA1*, which predisposes to early onset lung disease, specifically chronic obstructive pulmonary disease (COPD). In this issue of *PLOS Genetics*, the timely study of Thun *et al.* [1] investigated alpha one antitrypsin (AAT) levels with a genome-wide association study (GWAS) approach in a cohort recruited primarily for studies of asthma. They demonstrate that, although a genome-wide association is detected for a common variant in *SERPINA1*, the association disappears after adjusting the results for the presence of the known rare variants that cause AATD (PiZ and PiS). The observation of this “synthetic association” illustrates the potential for detection of rare genetic variants, with minor allele frequencies (MAF) of less than 1%, to revolutionize the understanding of pathogenesis.

Importantly, the approach used by Thun *et al.*—the sequencing of *SERPINA1*, fine mapping, and a conditional approach to statistical analysis in the regression model—does not depend on knowing the rare variants on which models need to be conditioned, and was able to reliably identify known variants with MAF 1%–5%. It is therefore a nice demonstration of the potential for sequencing to reveal rare variants and ascertain their true contribution to traits. Thun *et al.* have also compensated for known environmental exposure that interacts with genes, such as cigarette smoke exposure (“ever smoked”), enhancing this analysis by use of a proxy measure for smoke intensity (hsCRP). The robustness of the results was demonstrated using a second large cohort. The clinical relevance of this work was demonstrated by looking at lung function as the outcome, although this revealed a more complex scenario. The results leave some unanswered questions, such as the observation that variants influencing expression which lie outside *SERPINA1* exons are not accounted for, and that recent expression quantitative trait loci (eQTL) data for *SERPINA1* did not report their associated SNPs.

Since the advent of GWAS it has become increasingly obvious that this research design cannot detect the majority of the heritability of most studied traits. Several ideas have been proposed to explain this “missing heritability,” including gene–environment interaction, reduced power of GWAS to detect functional variants due to low levels of linkage disequilibrium with causative SNPs, and undetected rare variants. Thun *et al.* [1] provide an excellent realization of this last hypothesis. Most GWAS have picked up common variants that confer only a small measured increase in risk (increased odds ratios [ORs]), but have missed by design rare variants that confer larger ORs of disease. AATD and GWAS of COPD and lung function are perfect examples of this as they failed to detect both the PiZ and PiS variants, despite the well-established role of these mutations in lung disease. Only through a huge meta-analysis of over 20,000 individuals and smoking interaction modeling could the influence of PiZ [2] on lung function be detected. Thun *et al.* could use their analyses to begin to address the longstanding debate regarding the role of AATD in lung disease at the population level. Specifically, does carrying a single abnormal *SERPINA1* allele increase risk of lung disease at the population level [3]? A demonstration of an association, even at low AAT levels not considered truly deficient, would mean that AAT pathways are relevant to a far larger proportion of the population than previously thought. For this reason, the work of Thun *et al.* is not only an interesting example of synthetic association, but has potential clinical importance since it suggests that even small variation in AAT level could affect lung function [1].

**Figure 1:**
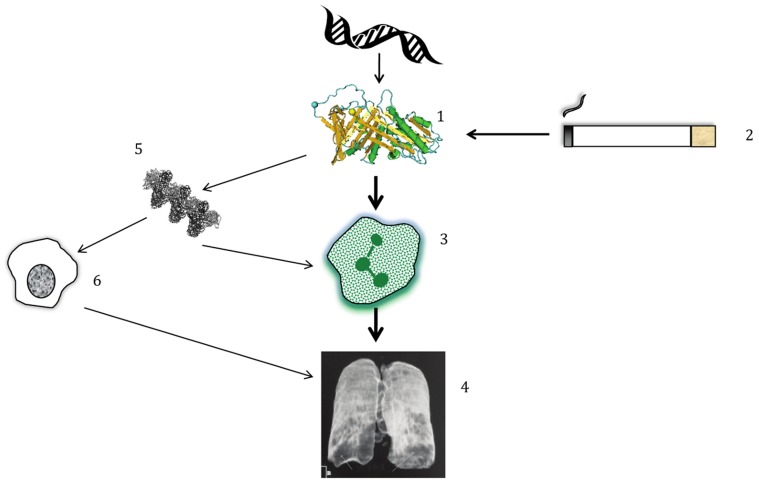
Simplified pathogenesis of lung disease in AATD. (1) Polymorphisms in DNA lead to structural changes in AAT which interact with (2) environmental exposure to cigarette smoke, amongst other influences. The combination of gene+environment leads to (3) neutrophilic inflammation in the lung; the main driver of disease is an inability to protect from the harmful effects of NE released by neutrophils. (4) Proteolytic destruction of lung tissue leads to the typical clinical pattern of emphysema, usually worst at the lung bases, as shown on this reconstructed image. (5 & 6) In addition, AAT polymers present in the lung, whose formation occurs due to the PiZ and to a lesser extent the PiS variant, play a smaller role in augmenting inflammation by attracting other inflammatory cells such as macrophages.

Since rare variant analysis is a topic of great interest to genetic epidemiologists at present, it is worth reprising how understanding AATD has moved clinical medicine forward. It was first described in 1963 by Laurell and Eriksson, who reported an absence of the α1 band on protein electrophoresis of serum taken from a patient at a local respiratory hospital [4]; this missing band was due to very low circulating levels of AAT. The observation that people with this deficiency developed early-onset emphysema and COPD suggested a role for pathways involving AAT in pathogenesis(summarized in Figure 1). The main function of AAT is as an anti-protease, which protects tissues against neutrophil elastase (NE) [5]; the protease–antiprotease hypothesis of emphysema resulted directly from this knowledge and has driven much of the research into COPD. Clinically significant AATD-related lung disease occurs in approximately 1 in 2,500 Caucasian individuals who usually carry the PiZ or PiS variants of *SERPINA1*; importantly, both SNPs confer an increased risk of lung disease in carriers who smoke and in homozygous individuals who do not smoke [6]. However, COPD is very common, affecting up to 20% of smokers and 11% of non-smokers worldwide, and is projected to be the third most common cause of death by 2030 [7]. Consequently, changing the direction of research in COPD had potential to greatly affect population health. At the time of detection of AATD, COPD as a term was not in common use; rather, patients were described as having emphysema or chronic bronchitis, both diagnosed by clinical features and chest radiography. It is now a common term and is diagnosed by a reduction in FEV1/FVC ratio on spirometry—a simple test of lung function—and may be further characterized by more complex lung function tests and computerized tomography (CT) scanning of the lungs.

Genetic research into the etiology of lung dysfunction in the general population [2] as well as the risk of COPD [8] has succeeded in identifying many variants that are significantly associated with these outcomes. Candidate gene approaches discovered other relevant proteases [9], and our increased understanding of the inflammatory cascade leading to neutrophil activation and NE release has guided the implementation of therapies targeting inflammation, the most common of which is inhaled corticosteroids (ICS). However, anti-inflammatory therapies have not been a magic bullet for COPD sufferers; indeed, the main trial seeking reduction in mortality showed only a trend in this direction (p = 0.052) when ICS were combined with long-acting beta agonists (LABA) [10]. However, lung function, flare-ups of the disease (known as exacerbations), and quality of life have been shown in many studies to improve with ICS/LABA combinations [7]. Relevant to the current paper on *SERPINA1* variants as a predictor of AAT, specific treatment for AATD has been developed in the form of AAT augmentation therapy. A meta-analysis of 1,509 patients in observational studies and randomized controlled trials (RCTs) concluded that it was beneficial, because decline in lung function was slower upon treatment [11]. More sophisticated measures of lung disease using quantitative analysis of CT scans have shown that augmentation is beneficial in the RCTs alone [12]. Whether or not the recognition that rare variants explain common variant associations at *SERPINA1* with AATD changes clinical practice remains to be seen, but the story should be a source of inspiration for genetic researchers to continue pursuing rare variants and their potential use in disease prediction and targeted therapy.
